# Longitudinal associations between bone and adipose tissue biochemical markers with bone mineralization in boys during puberty

**DOI:** 10.1186/s12887-016-0647-1

**Published:** 2016-07-20

**Authors:** Donvina Vaitkeviciute, Evelin Lätt, Jarek Mäestu, Toivo Jürimäe, Meeli Saar, Priit Purge, Katre Maasalu, Jaak Jürimäe

**Affiliations:** Institute of Sport Sciences and Physiotherapy, Centre of Behavioural, Social and Health Science, Faculty of Medicine, University of Tartu, Tartu, Estonia; Institute of Clinical Medicine, Faculty of Medicine, University of Tartu, Tartu, Estonia; Institute of Sport Pedagogy and Coaching Sciences, Centre of Behavioral, Social and Health Sciences, University of Tartu, 5 Jakobi Street, 51014 Tartu, Estonia

**Keywords:** Longitudinal analysis, Pubertal boys, Bone turnover, Adipokines, Physical activity

## Abstract

**Background:**

We investigated longitudinal relationships between the biochemical markers of bone and adipose tissue with bone mineral content (BMC), bone mineral density (BMD), moderate-to-vigorous physical activity (MVPA) and sedentary time (SED) in pubertal boys.

**Methods:**

Ninety-six boys (11.9 ± 0.6 years old) were measured at baseline, after 12 and 24 months. Body composition (fat mass [FM], lean body mass [LBM]), and whole body (WB), lumbar spine (LS) and femoral neck (FN) BMD and BMC were assessed. Additionally, serum leptin, adiponectin, osteocalcin (OC) and C-terminal telopeptide of type I collagen (CTX) were measured.

**Results:**

OC had a strong longitudinal inverse effect on changes in WB_BMD (*p* < 0.001) and LS_BMD (*p* = 0.021), while CTX had an inverse effect only on changes in FN_BMD (*p* = 0.011). Leptin had an inverse effect on changes in WB_BMC/WB_BMD (*p* = 0.001), FN_BMD (*p* = 0.002) and LS_BMD (*p* = 0.001). MVPA showed a longitudinal inverse effect on changes in leptin (*p* = 0.030), however no longitudinal effect of SED to biochemical markers of bone and adipose tissue was found.

**Conclusions:**

Bone metabolism markers have negative effect on bone mineral accrual during puberty. Increases in MVPA affect leptin, suggesting a positive link of MVPA through leptin metabolism on increases in bone mineralization during puberty.

## Background

Optimizing peak bone mass during puberty is a key factor for a healthy skeleton in later adult years [1], about 40 % of peak bone mass is accumulated during pubertal maturation [2]. Physical activity (PA) has been recommended as a possible prevention strategy against fragility fractures in older age [3]. Positive associations between PA and bone mineral density (BMD) during maturation have been reported in both, cross-sectional [4–6] and longitudinal [7] studies. Furthermore, PA has also been shown to influence bone turnover values in adolescents [8, 9].

Bone tissue remains metabolically active throughout the life; furthermore, during growth bone turnover markers reflect both modeling and remodeling of the current bone tissue [10]. However, bone metabolism markers are not site-specific and reflect the turnover of bone tissue of the whole skeleton [9]. There are uncertainties in the findings regarding osteocalcin (OC; biochemical marker of bone formation) and C-terminal telopeptide of type I collagen (CTX; biochemical marker of bone resorption) associations with bone mineral accrual during growth and maturation. Previous studies have reported negative associations between OC [11, 12], CTX [12] and bone mineral content (BMC) [11] or BMD [12] in male adolescents. Two studies have found no associations [13, 14] between OC [13, 14], CTX [14] and BMD, while one semi-longitudinal study of van Coeverden et al. [15] found positive correlation between OC and BMC values in 11–13.8 years old boys. However, longitudinal studies regarding bone formation and resorption markers take time effect also into account, which is important during sensitive periods of growth and maturation, especially during puberty. Previous studies have also used quite wide range of participants’ age [11–15] and used correlation coefficient as a statistical method to make conclusions [11–13; 15]. Furthermore, there is a strong lack of studies regarding the influence of PA on the association between bone turnover and BMD values in children during growth and maturation. One cross-sectional study with 8–17 year old children and adolescents reported that higher PA is associated with higher OC level [16]. In addition, a study with young adult males (25–30 years old) showed that active subjects had significantly higher serum OC level, compared with non-active males [17]. However, these studies are cross-sectional and long-term conclusions cannot be made. To our best knowledge, there are no longitudinal studies conducted that have reported the associations or effect of PA for CTX in healthy boys during puberty.

It is well known that adipose tissue produces different adipokines [18] that could mediate associations between bone and adipose tissue [19–21]. There are studies that have found no [22] or positive [19] associations between leptin and bone mineralization in male and female adolescents. A longitudinal study of Sayers et al. [23] showed an inverse association between adiponectin and bone mineral parameters in 9.9 to 15.5 year-old girls and boys. However, associations between adiponectin and bone mineral variables are less studied in boys, especially during puberty. Furthermore, the literature is also controversial about PA effect on adipokines, reporting no associations with adiponectin and leptin [24], or negative associations with leptin in girls, but not in boys [25]. However, the sample age differences and used different methods do not allow making conclusions.

Accordingly, the aim of the current study was to investigate the longitudinal associations of bone mineralization and PA level with bone and adipose tissue biochemical markers in healthy boys during puberty.

## Methods

### Subjects

In total, 96 boys aged 12–14 years old from different schools in Tartu (Estonia) took part in this longitudinal study. The boys were followed for 2 years and 3 measurement sessions were performed: at baseline, after 12 and after 24 months. The inclusion criteria for the current study were that a boy had to be healthy and allowed to take part in obligatory physical education lessons at school. The subjects were recruited from the schools with the information about the study was taken to all boys of the class. Those subjects who agreed, their parents received detailed information about the study and the signed informed consent was obtained from parents, while children gave the verbal assent. The subjects had the right to withdraw from the study at any time.

### Anthropometric measurements

Body height (cm) was measured using Martin’s metal anthropometer to the nearest 0.1 cm. Body mass (kg) was measured to the nearest 0.05 kg using medical scales (A&D Instruments, Abingdon, UK). Body height and body mass data were used to calculate body mass index (BMI; kg/m^2^).

### Pubertal development

Pubertal development was assessed by self-report questionnaire of pubertal stages according to Tanner [26]. Boys were provided with line drawings, pictures and descriptions representing genitalia and pubic hair development stages. The subjects were asked to choose the appropriate development stage by themselves. In the case of discrepancies between the two variables, greater emphasis for determination of the Tanner stage was placed on the degree of genitalia development [27]. The pubertal stage assessment according to Tanner method, which uses the self-assessment of genitalia and pubic hair stage, has been previously validated [28, 29].

### Bone mineral parameters and body composition

Whole-body (WB) BMD and BMC, fat mass (FM) and lean body mass (LBM) were measured. BMD was also measured at the skeletal sites of lumbar spine (LS) and femoral neck (FN). Dual-energy X-ray absorptiometry (*DXA; DPX-IQ densitometer*, Lunar Corporation, Madison, USA) equipped with proprietary software (version 3.6) was used for all body composition parameter measurements. The densitometry procedure has been described in our previous study [28].

### Physical activity

A uniaxial accelerometers GT1M (ActiGraph, Pensacola, USA) were used to assess PA and have been previously validated in children and adolescents [30]. All participants wore the accelerometer for 7 consecutive days. Boys were instructed to remove the devices during water-based activities and during sleep period. The interval of time (epoch) was set at 15 s. At least two weekdays and one weekend day of recording with a minimum of 8 h/day was set as an inclusion criterion [31]. The following cut-offs were used: sedentary time (SED; < 100 counts/min), moderate PA (MPA; 2000–4000 counts/min) and vigorous PA (VPA; > 4000 counts/min) [32]. Moderate-to-vigorous PA (MVPA; ≥ 2000 counts/min) was calculated as the sum of moderate and vigorous PA.

### Blood analysis

A 10 mL blood sample was obtained from an antecubital vein with the participant sitting in the upright position after an overnight fast between 07.30 and - 08.30 h. The blood serum was separated and then frozen at −80 °C for further analysis. Leptin was determined by radioimmunoassay (RIA) (Mediagnost, Reutlingen, Germany). This assay has intra- and interassay CVs less than 5 %, and the least detection limit was 0.01 ng/ml. Total adiponectin was also determined with a commercially available RIA kit (cat. no. HADP-61HK; Linco Research, St. Charles, MO, USA). The intra- and interassay CVs were less than 7 %, and the least detection limit was 1 μg/ml. Total OC and CTX were analysed using Immulite 2000 (DPC, Los Angeles, USA). The intra- and interassay CVs for OC and CTX were less than 7 %.

### Data analysis

All statistical analyses were performed using SPSS software version 21.0 (SPSS Inc.) and SAS 9.2 (SAS Institute, Inc. Cary, NC, USA). Standard statistical methods were used to calculate means and standard deviations (± SD). Shapiro-Wilks test and q-q plots controlled normality of parameters. Analysis of variance (ANOVA) for repeated measures was used to determine significant differences between three measurement sessions (baseline, after 12 and after 24 months). For longitudinal analyses, multilevel fixed effects regression models were constructed using PROC MIXED method (SAS version 9.2). Multilevel modeling allowed us to include participants who randomly missed some of the measurements. The coefficients of fixed variables were used to predict WB_BMC/BMD, FN_BMD and LS_BMD. Longitudinal models were controlled by chronological age, BMI and Tanner stage. *P*-values less than 0.05 were considered to indicate statistical significance.

## Results

Mean pubertal stage, height, body mass, BMI, FM, LBM and all bone mineral parameters increased significantly during every measurement session (Table 1). Adiponectin concentration increased significantly after 12 months of the study, however it decreased significantly after 24 months showing the peak level during the second measurement session. OC and CTX also increased significantly over the first 12 months of the study and remained significantly higher after 24 months compared with baseline level. MVPA decreased over the 24-month study period with significant decrement between baseline and after 24 months measurements. SED has been increasing during the study period with significant increment between baseline and after 24 months measurements.

**Table 1:** Mean (± SD) characteristics of the subjects

Variable	At baseline (*n* = 96)	After 12 months (*n* = 96)	After 24 months (*n* = 96)
Age (years)	11.9 ± 0.6	12.9 ± 0.6^a^	13.9 ± 0.6^a,b^
Tanner stage 1/2/3/4/5	2.7 ± 0.6 0/40/48/8/0	3.3 ± 0.9^a^ 0/17/47/22/10	3.9 ± 0.8^a,b^ 0/3/24/42/25
Height (cm)	153.8 ± 7.4	161.5 ± 8.4^a^	168.3 ± 8.3^a,b^
Body mass (kg)	49.3 ± 16.0	55.9 ± 17.7^a^	62.1 ± 18.8^a,b^
BMI (kg/m^2^)	20.5 ± 5.2	21.1 ± 5.3^a^	21.7 ± 5.3^a,b^
Fat mass (kg)	13.4 ± 10.5	14.7 ± 11.2^a^	15.1 ± 11.7^a^
Lean body mass (kg)	33.2 ± 6.5	38.5 ± 8.3^a^	43.7 ± 9.4^a,b^
WB_BMC (g)	1721.1 ± 384.5	1986.7 ± 474.4^a^	2265.9 ± 533.9^a,b^
WB_BMD (g/cm^2^)	0.983 ± 0.069	1.018 ± 0.081^a^	1.060 ± 0.098^a,b^
FN _BMD (g/cm^2^)	0.895 ± 0.086	0.940 ± 0.103^a^	0.985 ± 0.120^a,b^
LS _BMD (g/cm^2^)	0.831 ± 0.097	0.890 ± 0.121^a^	0.966 ± 0.147^a,b^
Leptin (ng/ml)	7.1 ± 8.3	6.3 ± 7.2	5.4 ± 7.3
Adiponectin (μg/ml)	8.9 ± 4.7	10.5 ± 4.7^a^	8.3 ± 4.4^b^
Osteocalcin (ng/ml)	110.4 ± 37.6	136.4 ± 48.0^a^	132.1 ± 44.6^a^
CTX (ng/ml)	1.6 ± 0.4	1.9 ± 0.5^a^	1.9 ± 0.5^a^
MVPA (min/day)	64.5 ± 23.5	60.4 ± 25.8	56.5 ± 26.1^a^
SED (min/day)	537.2 ± 72.7	556.4 ± 78.8	574.0 ± 93.2^a^

*BMI* body mass index, *WB* whole body, *LS* lumbar spine, *FN* femoral neck, *BMD* bone mineral density, *BMC* bone mineral content, *CTX* C-terminal telopeptide of type I collagen, *MVPA* moderate-to-vigorous physical activity, *SED* sedentary time

^a^Significant changes from baseline

^b^Significant changes after 12 months

Multilevel regression models indicated that subjects varied significantly at each measurement occasion in their level of WB_BMC, WB_BMD and FN BMD (Table 2). OC had the significant inverse effect for explanation WB_BMD (*p* < 0.001) and LS_BMD (*p* = 0.021) changes in pubertal boys. However, the effect of CTX was inversely significant only for FN_BMD change (*p* = 0.011). Leptin had a significant negative effect in the explanation of WB_BMD/BMC (*p* = 0.001), FN_BMD (*p* = 0.002) and LS_BMD (*p* = 0.001) changes, while the effect of adiponectin was not significant in the models. Results from Table 3 indicated that MVPA had a negative effect in the explanation for leptin (*p* = 0.030) changes. No effect of MVPA to OC, CTX or adiponectin changes were found. Additionally, it was found that SED had no longitudinal effect to OC, CTX, leptin and adiponectin in boys during puberty (Table 3).

**Table 2:** Multilevel regression models for variables that contribute to the change in WB_BMC, WB_BMD, FN_BMD and LS_BMD

Variables	WB_BMC		WB_BMD		FN_BMD		LS_BMD	
Fixed effect	Estimates ± SE	*P* value	Estimates ± SE	*P* value	Estimates ± SE	*P* value	Estimates ± SE	*P* value
Intercept	−1669.01 ± 140.83	<0.001	0.4690 ± 0.0267	<0.001	0.3354 ± 0.0508	<0.001	0.0754 ± 0.058	0.198
Osteocalcin	−0.6044 ± 0.3273	0.069	−0.0003 ± 0.0001	<0.001	0.00004 ± 0.0001	0.743	−0.0003 ± 0.0001	0.021
CTX	7.6320 ± 32.3889	0.814	0.0014 ± 0.0063	0.827	−0.0307 ± 0.0118	0.011	−0.0155 ± 0.0125	0.219
Leptin	−9.9625 ± 2.5970	0.001	−0.0019 ± 0.0005	0.001	−0.0031 ± 0.0010	0.002	−0.0037 ± 0.0010	0.001
Adiponectin	2.7653 ± 1.9866	0.168	0.0006 ± 0.0004	0.138	0.0010 ± 0.0007	0.173	0.0004 ± 0.0008	0.587

*SE* standard error, *BMI* body mass index, *WB* whole body, *LS* lumbar spine, *FN* femoral neck, *BMD* bone mineral density, *BMC* bone mineral content, *CTX* C-terminal telopeptide of type I collagen, *MVPA* moderate-to-vigorous physical activity, Model was controlled by age, BMI and Tanner stage

**Table 3:** Multilevel regression models for variables that contribute to the change in osteocalcin, CTX, leptin and adiponectin

Variables	Osteocalcin		CTX		Leptin		Adiponectin	
Fixed effect	Estimates ± SE	*P* value	Estimates ± SE	*P* value	Estimates ± SE	*P* value	Estimates ± SE	*P* value
Intercept	6.713 ± 39.312	0.865	0.413 ± 0.391	0.294	1.304 ± 3.500	0.710	20.724 ± 3.725	<0.001
MVPA	−0.0368 ± 0.1095	0.738	−0.0012 ± 0.0011	0.304	−0.0219 ± 0.099	0.030	−0.01942 ± 0.0114	0.093
Intercept	−16.886 ± 38.968	0.658	0.1384 ± 0.377	0.715	−3.0598 ± 3.522	0.387	16.109 ± 3.600	<0.001
SED	0.0611 ± 0.0324	0.063	0.0004 ± 0.0003	0.189	0.0051 ± 0.0029	0.079	0.0066 ± 0.0033	0.052

*SE* standard error, *BMI* body mass index, *CTX* C-terminal telopeptide of type I collagen, *MVPA* moderate-to-vigorous physical activity, *SED* sedentary time, Model was controlled by age, BMI and Tanner stage

## Discussion

The important finding of this study was that bone formation marker OC had an inverse longitudinal effect on changes in WB_BMD/BMC (*p* < 0.001) and LS_BMD (*p* = 0.021). Moreover, we found that bone resorption marker CTX had an inverse longitudinal effect on changes in FN_BMD (*p* = 0.011). To our best knowledge, there are no longitudinal studies regarding the associations between bone metabolism markers and bone mineral accrual in boys during puberty.

Few cross-sectional studies have reported positive associations between OC and BMC values [15], negative associations between OC and BMD [11, 12], and no associations between OC [13, 14] or CTX [14] and bone mineral values. There is only one five-year longitudinal study that was conducted with young men (18.9 years old at baseline) [33] and reported that OC predicts increment in BMD and BMC of WB, LS and radius.

According to our longitudinal results, it could be suggested that bone formation marker (OC) and bone resorption marker (CTX) negatively affect bone mineralization in healthy pubertal boys. The different results between our longitudinal study and that of Darelid et al. [33] could be explained by the different age range of studied males. It has been suggested previously that while bone turnover markers are higher in early puberty, greater bone mineral accrual occurs later puberty when markers are declining [10, 17]. Specifically, it has been reported that lower concentrations of bone formation and resorption markers predict increased BMD values in children during the development of peak skeletal mass [10]. Cross-sectional study with 12.5–17.5 year-old adolescents supports our findings and reports the lower bone formation and resorption markers in older adolescents compared to younger ones [34]. Furthermore, decreasing bone turnover in adolescence results in longer mineralization of bone tissue during early adulthood [17]. Finally, it is important to measure more than one bone formation and resorption markers together with densitometric parameters to better detect effects in bone growth [17]. Accordingly, further longitudinal studies during growth and early adulthood are needed to characterize the influence of specific bone turnover markers in bone mineral acquisition.

Multilevel regression indicated that leptin has inverse longitudinal effect in the explanation of increases in WB_BMC/BMD (*p* = 0.001), FN_BMD (*p* = 0.002) and LS_BMD (*p* = 0.001). In contrast, adiponectin had no longitudinal effect in the explanation of WB_BMC/BMD, FN_BMD and LS_BMD changes in healthy boys during puberty. While the association between leptin and bone mineral values has previously been studied in children [19,22; 35–36], there is only one longitudinal research about the associations between adiponectin and bone mineralization that reported an inverse association between adiponectin and bone mineral parameters in children [23]. However, our findings of associations between leptin and bone mineralization are similar to the results of Prado et al. [35] study, where inverse associations between leptin and BMD were found in 13–18 years old boys. Other cross-sectional studies have reported positive [36] or no [22] associations between leptin and WB or regional BMD and BMC in adolescent boys. However, these studies are cross-sectional and carried with relatively small sample size [22] or with prepubertal children [36]. Girls are more studied than boys [37, 38], however our study does not confirm the findings of the study with pubertal girls where no relationship between leptin and WB_BMC and WB_BMD were found [38]. Furthermore, according to our results, it can be argued that FM, which is directly correlated with leptin in healthy subjects [39], has an inverse longitudinal effect for bone mineralization in boys during puberty. These results are in accordance with a cross-sectional study with 6-year old children, which concluded that FM is negatively associated with volumetric bone density independent of LBM [40]. However, a cross-sectional study with similar age range subjects (12.5–17.5 years) to our study concluded that FM is positively associated with bone mass independent of LBM, but after controlling for LBM, the associations between FM and bone mass became inverse [41]. Also a study with 7–19 years old boys and girls concluded that low or high body FM could influence skeletal development, while normal level of FM is necessary for bone health in growing children [42]. Taken together, our longitudinal study suggests that leptin but not adiponectin significantly affects changes in bone mineral parameters (BMC and BMD) in boys during puberty.

The role of PA impact on bone mineralization during growth and maturation is well known [4–7] and the decrease in MVPA and increase in SED during puberty has been reported before [43]. Increased SED among pubertal boys is concerning knowing that SED was found to have negative influence on whole body bone mass in growing adolescents [44]. Our longitudinal study also reports the significant decrease in MVPA and significant increase in SED over the 24-month period in boys during puberty.

The effect of PA on bone turnover markers during pubertal maturation is highly understudied [8–9, 16]. While the association between PA and OC has been studied in young adult males [17], to our best knowledge, there are no studies that reported the longitudinal associations of the effect of PA on CTX in adolescents. While a cross-sectional study with 8–17 year old children and adolescents reported that higher PA is associated with higher OC level [16], our longitudinal study did not find the longitudinal effect of MVPA or SED to OC after controlling for age, BMI and pubertal stage. Such disagreement could be a result of different study design (cross-sectional versus longitudinal), applied PA measurement methods (questionnaire versus accelerometer) and sample age, whereas we conducted a longitudinal study with objectively measured PA and our sample age was very narrow during puberty. In addition, our longitudinal analysis showed that MVPA and SED did not influence changes in CTX in boys during puberty. However, further longitudinal studies with adolescents are necessary to look for a longitudinal effect of habitual or special physical training exercises on changes in bone turnover markers during puberty.

In agreement with results of a longitudinal study with 5-year old boys at baseline [24], our study found no associations between objectively measured MVPA and adiponectin in boys during puberty. However, we found a significant longitudinal effect of MVPA for leptin (*p* = 0.030). Such results were highly expected as it is known that PA level is associated with lower FM in children and adolescents [45]. The influence of PA on leptin level in children and adolescents is controversial. Study of Romon et al. [25] found no associations between leptin and PA in a group of 8–18 years old males; while in females such associations were found, however, the PA was not measured objectively, authors used questionnaires and sample size was with a wide range in age. Our study supports the results of Jiménez-Pavón et al. [46], which reported that PA decreases the level of leptin circulation in 12.5–17.5 years old male and females. Accordingly, our longitudinal study reports that MVPA has an effect in the explanation of leptin, suggesting that MVPA, but not SED is an important factor that reduces the excess amount of FM and further reduces the negative influence of FM on bones in healthy pubertal boys.

The major strength of our study is a longitudinal design (24-month observation period with 3 measurement sessions); other strengths of the current study are the measured biochemical markers of bone formation (OC), resorption (CTX) and adipokines (leptin, adiponectin). The use of accelerometer with 15 s epochs to objectively monitor PA level and the use of DXA add more strength to the present study. However, a relatively small sample size (96 pubertal boys) is a major limitation to our study.

## Conclusions

Bone formation (OC) and bone resorption (CTX) markers negatively affect bone mineralization in healthy boys during puberty. Leptin, but not adiponectin was inversely associated with BMC and BMD increment. Finally, MVPA negatively influenced the leptin level in pubertal boys indicating a strong effect against adolescents’ obesity problem.

## Abbreviations

BMC, bone mineral content; BMD, bone mineral density; BMI, body mass index; CTX, C-terminal telopeptide of type I collagen; CV, coefficient of variation; DXA, dual-energy X-ray absorptiometry; FM, fat mass; FN, femoral neck; LBM, lean body mass; LS, lumbar spine; MVPA, moderate-to-vigorous physical activity; OC, osteocalcin; PA, physical activity; SD, standard deviation; SE, standard error; SED, sedentary time; WB, whole body
